# Mitochondrial Quality Control and Cellular Proteostasis: Two Sides of the Same Coin

**DOI:** 10.3389/fphys.2020.00515

**Published:** 2020-05-25

**Authors:** Justin M. Quiles, Åsa B. Gustafsson

**Affiliations:** Department of Pharmacology, Skaggs School of Pharmacy and Pharmaceutical Sciences, University of California, San Diego, La Jolla, CA, United States

**Keywords:** mitochondria, UPS, proteasome, UPR, proteotoxicity, import, mitophagy, Parkin

## Abstract

Mitochondrial dysfunction is a hallmark of cardiac pathophysiology. Defects in mitochondrial performance disrupt contractile function, overwhelm myocytes with reactive oxygen species (ROS), and transform these cellular powerhouses into pro-death organelles. Thus, quality control (QC) pathways aimed at identifying and removing damaged mitochondrial proteins, components, or entire mitochondria are crucial processes in post-mitotic cells such as cardiac myocytes. Almost all of the mitochondrial proteins are encoded by the nuclear genome and the trafficking of these nuclear-encoded proteins necessitates significant cross-talk with the cytosolic protein QC machinery to ensure that only functional proteins are delivered to the mitochondria. Within the organelle, mitochondria contain their own protein QC system consisting of chaperones and proteases. This system represents another level of QC to promote mitochondrial protein folding and prevent aggregation. If this system is overwhelmed, a conserved transcriptional response known as the mitochondrial unfolded protein response is activated to increase the expression of proteins involved in restoring mitochondrial proteostasis. If the mitochondrion is beyond repair, the entire organelle must be removed before it becomes cytotoxic and causes cellular damage. Recent evidence has also uncovered mitochondria as participants in cytosolic protein QC where misfolded cytosolic proteins can be imported and degraded inside mitochondria. However, this process also places increased pressure on mitochondrial QC pathways to ensure that the imported proteins do not cause mitochondrial dysfunction. This review is focused on discussing the pathways involved in regulating mitochondrial QC and their relationship to cellular proteostasis and mitochondrial health in the heart.

## Introduction

Mitochondrial dysfunction is a hallmark of cardiac pathophysiology. Defects in mitochondrial performance disrupt contractile function, overwhelm myocytes with reactive oxygen species (ROS), and transform these cellular powerhouses into pro-death organelles (Zhou and Tian, 2018). Accordingly, quality control (QC) pathways aimed at identifying and removing damaged mitochondrial proteins, components, or entire mitochondria represent crucial adaptive responses for cardiac myocytes. Because the majority of the mitochondrial proteins are encoded by the nucleus and translated in the cytosol, cytosolic protein QC mechanisms are intimately linked with mitochondrial fidelity. The cytosolic ubiquitin proteasome system (UPS) ensures that functional proteins are delivered to the mitochondria (Bragoszewski et al., 2013; Wang and Chen, 2015). Thus, it is not surprising that perturbations to protein homeostasis or proteostasis during cardiac pathophysiology (Hofmann et al., 2019) also antagonize mitochondrial function and activate cell death pathways in cardiac myocytes (Zhou and Tian, 2018).

Mitochondria contain their own protein QC system to prevent damaged or misfolded proteins from accumulating. There are chaperones that assist with folding the newly imported proteins, as well as proteases that cleave misfolded or non-functional proteins (Szczepanowska et al., 2016; Weinhäupl et al., 2018). However, if levels of misfolded proteins exceed the folding and degradative capacity of the resident chaperones and proteases, activation of the mitochondrial unfolded protein response (UPR^mt^) will ensue. This conserved response involves retrograde signaling to the nucleus to activate a transcriptional program aimed at restoring mitochondrial proteostasis (Münch and Harper, 2016). Intriguingly, recent reports suggest that mitochondria themselves can contribute to cytosolic protein QC through the import and degradation of misfolded proteins in the matrix (Burman et al., 2017; Ruan et al., 2017; Li et al., 2019). Thus, while mitochondria require cytosolic protein QC mechanisms for proper structure, these organelles directly promote cytosolic proteostasis during proteotoxic stress. However, at a certain threshold, the damage may become too great for protein repair and the entire mitochondrion must be eliminated from the cell. The primary mechanism by which entire mitochondria are eliminated is via autophagy and involves engulfment of the organelle into autophagosomes. In this review, we discuss the pathways involved in regulating mitochondrial QC and their relationship to cellular proteostasis and mitochondrial health in the heart.

## Regulation of Mitochondrial Quality by the Ubiquitin Proteasome System (UPS)

The UPS is essential for routine protein turnover as well as the degradation of misfolded and unfolded proteins. Even modest decreases in proteasomal efficiency sensitize mice to cardiac pathogenesis (Ranek et al., 2015). Ventricular biopsies from human patients have been shown to exhibit decreased proteasomal activity and increased levels of protein ubiquitination (Predmore et al., 2010). The UPS is also involved in degrading accessible proteins in the outer mitochondrial membrane (OMM) with downstream effects on mitochondrial morphology and apoptosis. For example, UPS-mediated degradation of the anti-apoptotic protein MCL-1 allows for activation of pro-death proteins Bax/Bak (Zhong et al., 2005), while degradation of the mitochondrial fusion protein Mitofusin 2 leads to a shift toward fragmented mitochondria and disconnection from the endoplasmic reticulum (McLelland et al., 2018). After ubiquitination, OMM proteins are extracted from the membrane and delivered to the proteasome, a pathway analogous to the degradation of endoplasmic reticulum cargo during protein misfolding. The AAA-ATPase p97 is required for the extraction of specific substrates such as MCL-1 (Xu et al., 2011) and the general degradation of oxidized OMM proteins (Hemion et al., 2014). Studies employing a dominant negative p97 indicate that this QC pathway is critical to maintain a mitochondrial membrane potential (Fang et al., 2015). In addition to the outer membrane, proteins within the intermembrane space can also be exported through the translocase of the outer mitochondrial membrane (TOM) complex for proteasomal degradation. These QC mechanisms provide a means for the turnover of specific mitochondrial resident proteins and have been reviewed elsewhere (Karbowski and Youle, 2011).

**FIGURE 1:**
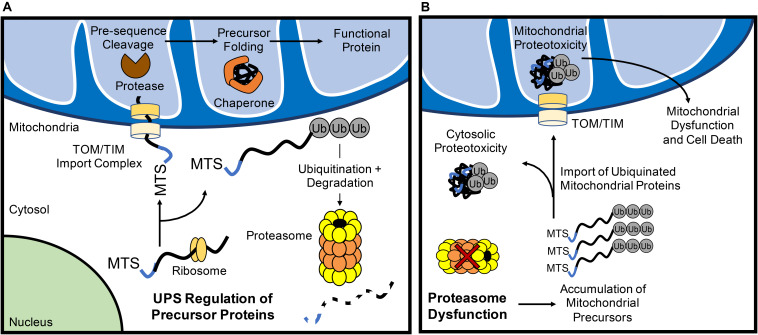
The ubiquitin proteasome system (UPS) regulates mitochondrial protein turnover. **(A)** The UPS monitors mitochondrial precursor proteins before they are imported into mitochondria. After import via the TOM/TIM complex in an unfolded state, the mitochondrial targeting sequence (MTS) is cleaved off by a protease and chaperones ensure proposer folding of the protein. **(B)** Inhibition of UPS results in the accumulation of polyubiquinated mitochondrial precursor proteins in the cytosol. Some of the ubiquitinated proteins are still imported into the mitochondrion which can contribute to mitochondrial dysfunction and activation of cell death.

More recently, the UPS has emerged as an important mechanism in maintaining mitochondrial QC through the turnover of nuclear-encoded mitochondrial proteins prior to their import. All but 13 of the >1000 mitochondrial proteins are nuclear-encoded and need to be transported to the mitochondrion where they are subsequently imported through the TOM/TIM complexes in an unfolded state. As such, the UPS provides the first line of mitochondrial QC through its consistent surveilling of mitochondrial proteins during their translation (Figure 1A). Increases in subunits of respiratory complexes I, II, and IV, as well as the F1-F0-ATPase upon acute proteasomal inhibition indicate that many of these proteins are quickly degraded by the UPS before they even reach their subcellular localization in the mitochondrial matrix (Margineantu et al., 2007). Similar increases in endonuclease G (Radke et al., 2008) and UCP2 (Azzu and Brand, 2010), proteins known to reside in the intermembrane space and inner membrane, respectively, upon UPS inhibition suggest that most precursor proteins routed to the inner mitochondrial sub-compartments are subject to this level of QC. The ubiquillin family of proteins have recently emerged as a key link between mitochondrial precursor protein targeting and UPS monitoring. In the cytosol, ubiquillins specifically interact with mitochondrial transmembrane proteins to facilitate their processing and membrane insertion (Itakura et al., 2016). In the event of failed insertion, ubiquillins recruit an E3 ligase for the polyubiquitination and proteasomal degradation of transmembrane domain-containing mitochondrial proteins. Mammals possess four ubquillin proteins with seemingly redundant functions in mitochondrial protein QC (Itakura et al., 2016). Interestingly, ubiquillin1 was recently shown to be necessary for myocardial proteostasis. Cardiac-restricted ubiquillin1 knockout (KO) mice develop cardiac dysfunction by 5 months of age and die prematurely (Hu et al., 2018). Moreover, at 10 weeks of age, ubiquillin1-deficient mice subjected to ischemia-reperfusion (I/R) injury have significantly greater cardiac dysfunction and infarct size compared to age-matched wild type mice, suggesting that ubiquillin1 plays an important role in the adaptation to myocardial stress (Hu et al., 2018). However, whether loss of ubiquillin1 in myocytes leads to accumulation of mitochondrial transmembrane proteins in the cytosol and its effect on mitochondrial function still need to be investigated.

If the UPS continuously monitors mitochondrial precursor protein localization, what are the fates of these proteins when proteasomal activity is inhibited? Investigations into this question have yielded varying results that likely relate to the structure and import efficiency of particular mitochondrial proteins. For example, pharmacological inhibition of the proteasome in HeLa cells leads to a significant increase in the levels of ubiquitinated proteins in the inner mitochondrial membrane (Lavie et al., 2018), suggesting that ubiquitinated proteins are imported into mitochondria (Figure 1B). Li et al. (2019) also found that inhibition of the proteasome leads to increased import of proteins into mitochondria. In contrast, a recent study in Neuro-2a cells revealed that inhibiting the UPS leads to the irreversible aggregation of respiratory complex subunits in the cytosol (Rawat et al., 2019) (Figure 1B). In this report, several electron transport chain proteins were found to accumulate in insoluble protein fractions which leads to increased oxidative stress and respiratory defects (Rawat et al., 2019). Similarly, disruption in various mitochondrial functions including inhibited ATP/ADP exchange across the inner membrane (Wang and Chen, 2015) and impaired mitochondrial translation (Fakruddin et al., 2018) leads to mitochondrial protein aggregation in the cytosol. Cytosolic aggregation of mitochondrial proteins can occur downstream of import defects, resulting in a compensatory attenuation of cytosolic translation and concomitant increase in proteasomal activity to process mistargeted precursor proteins (Wrobel et al., 2015). Although these responses transiently increase protein QC (Wrobel et al., 2015), the sustained accumulation of the precursor proteins leads to cell death (Wang and Chen, 2015). Thus, it is clear that a dysfunctional UPS results in accumulation of ubiquinated mitochondrial protein aggregates in both the cytosolic and mitochondrial compartments of the cell. Taken together, this illustrates the importance of proper trafficking and import of nuclear-encoded mitochondrial proteins for cytosolic proteostasis.

## Mitochondrial Unfolded Protein Response (UPR^mt^)

**FIGURE 2:**
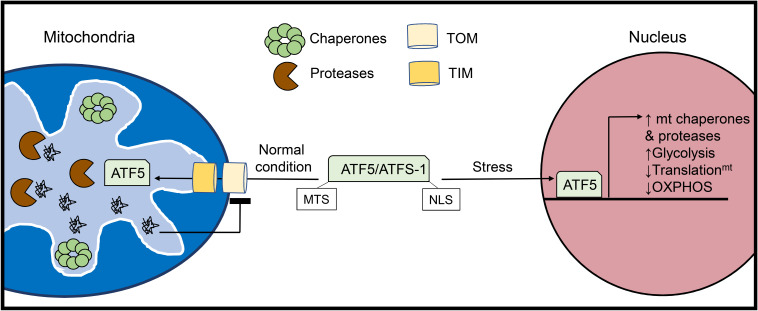
Activation of the mitochondrial unfolded protein response (UPR^mt^). The UPR^mt^ is activated when there is an excess of misfolded proteins within the organelle. The UPR^mt^ transcriptional regulator ATF5 (ATFS-1 in yeast) contains both a mitochondrial targeting sequence (MTS) and a nuclear localization signal (NLS). Under normal conditions, ATF5 is imported into mitochondria where it is degraded. However, increased levels of misfolded proteins inside mitochondria lead to abrogation of ATF5 import. Instead, ATF5 translocates to the nucleus where it activates a transcriptional response aimed at restoring mitochondrial health and proteostasis.

Because the majority of mitochondrial proteins are nuclear-encoded and imported in an unfolded state, mitochondria contain resident chaperones and proteases to ensure proper protein folding and degradation of aberrant precursor molecules. The proteases in the matrix are also responsible for the normal turnover of proteins inside the mitochondria (Bulteau et al., 2017). LonP1 is the most abundant protease in cardiac mitochondria (Bota and Davies, 2002; Lau et al., 2012) and is responsible for degrading various misfolded and oxidatively damaged matrix and inner membrane proteins, thereby preventing their deleterious accumulation (Bota and Davies, 2002). When the levels of misfolded proteins exceed the folding and degradative capacity of the resident chaperones and proteases, a conserved mitochondrial unfolded protein response (UPR^mt^) is activated (Figure 2). This involves retrograde signaling to the nucleus to promote a transcriptional program aimed at restoring mitochondrial proteostasis through the induction of chaperones and proteases (Münch and Harper, 2016). Similar to the endoplasmic reticulum UPR (UPR^ER^), the UPR^mt^ is activated by an accumulation of misfolded proteins and results in nuclear transcription of proteostatic genes. However, the UPR^mt^ can be distinguished from the UPR^ER^ in the downstream transcriptional targets. For example, the UPR^ER^ induces the expression of resident ER chaperones Calreticulin (*CALR*), GRP78/BiP (*HSPA5*), GRP94 (*HSP90B1*), which harbor the ER-targeting peptide sequence KDEL, to promote protein folding in the ER lumen (Yamamoto et al., 2003). For a more comprehensive understanding of ER-stress signaling and its relationship to cardiovascular disease, readers are referred to a recent review (Amen et al., 2019).

The UPR specific to the mitochondria was initially discovered in cultured mammalian cells through overexpression of the truncated mitochondrial matrix protein ornithine transcarbamylase (OTC-Δ) which is prone to aggregation. This study found that overexpression of OTC-Δ and the resulting mitochondrial stress trigger increased expression of the mitochondrial chaperones HSP60 (*HSPD1*) and mtDNAJ (*DNAJ3*), as well as the matrix protease CLPP via the transcription factor CHOP (*DDIT3*) (Zhao et al., 2002). The UPR^mt^ also upregulates glycolysis while downregulating subunits in the respiratory chain and mitochondrial translation to unburden the mitochondria while proteostasis is restored (Nargund et al., 2012, 2015). In parallel, the UPR^mt^ promotes the expression of various genes responsible for mitochondrial protein import, OXPHOS assembly, and mitochondrial ROS detoxification (Shpilka and Haynes, 2018) (Figure 2). In addition to intraorganellar proteotoxicity, the UPR^mt^ has emerged as a multifaceted response to several mitochondrial stressors including mito-nuclear protein imbalance, import defects, and mtDNA depletion. As such, the transcriptional regulators and downstream effectors are currently under intense investigation. In mammalian cells, activating transcription factor 5 (ATF5) is a major regulator of UPR^mt^ activation and possesses both a mitochondrial translocation sequence and a nuclear localization signal (Fiorese et al., 2016). In the basal state, ATF5 is imported into mitochondria and subsequently degraded by resident proteases. UPR^mt^ activation disrupts the mitochondrial import of ATF5 enabling its nuclear translocation and activation of downstream effector genes. ATF5 has been reported to induce a variety of genes involved in the UPR^mt^, including HSP60, mtHSP70, and the matrix protease LonP1 (Fiorese et al., 2016). In addition to ATF5, CHOP (Aldridge et al., 2007) and ATF4 (Quirós et al., 2017), two known transcriptional regulators of the UPR^ER^ (Amen et al., 2019) have been identified as mediators of the UPR^mt^, although it is currently unclear how these transcription factors can orchestrate distinct proteostatic responses in the ER and mitochondria.

Activation of the UPR^mt^ has been reported to be important in myocytes. Inhibition of the mitochondrial chaperone HSP90 or OXPHOS Complex I in neonatal rat myocytes leads to activation of a canonical UPR^mt^ transcriptional response including the induction of ATF5, CHOP, HSP60, mtDNAJ, LonP1, and ClpP (Smyrnias et al., 2019). The authors confirmed that overexpression of the aggregate prone mitochondrial OTC-Δ in myocytes leads to a similar transcriptional profile. These genes are also increased in a small cohort of human patients undergoing valve replacement for aortic stenosis. Interestingly, a subset of patients with strong induction of UPR^mt^ exhibit reduced levels of serum markers for myocardial damage, as well as fewer apoptotic myocytes and fibrosis in tissue sections relative to patients with low induction of UPR^mt^ (Smyrnias et al., 2019). These clinical observations indicate that a robust UPR^mt^ response in cardiac myocytes might protect the heart against pathological remodeling and dysfunction. Indeed, pharmacological activation of the UPR^mt^ preserves ejection fraction and reduces infarct size in murine models of pressure overload (Smyrnias et al., 2019) and I/R injury (Wang et al., 2019), respectively. Importantly, ATF5 is required for the cardioprotective effects of the UPR^mt^ in these studies (Smyrnias et al., 2019; Wang et al., 2019). This is in contrast to the UPR^ER^ which is primarily regulated by ATF6 in the heart (Jin et al., 2017; Blackwood et al., 2019). Consistent with the need for transcriptional activation, acute activation of the UPR^mt^ is insufficient to protect against I/R injury (Wang et al., 2019). Furthermore, while UPR^mt^ activation preserves cardiac and mitochondrial functions, the hypertrophy response is not abrogated suggesting that early responses in cardiac growth occur independent of mitochondrial impairment (Smyrnias et al., 2019).

Current knowledge of the molecular mechanism of UPR^mt^-mediated protection during cardiac pathophysiology is limited. In addition to ATF5, Hsp60 and LonP1 that function downstream also appear to be essential in cardiac myocytes for protection against stress. Deletion of Hsp60 in adult cardiomyocytes produces a lethal cardiomyopathy within weeks of tamoxifen-mediated gene deletion. Interestingly, although a UPR^mt^ response occurred following the loss of Hsp60, it is not sufficient to restore mitochondrial homeostasis suggesting a key role for Hsp60 in this response (Fan et al., 2019). Similarly, the LonP1 protease is critical for OXPHOS turnover during myocardial ischemia (Sepuri et al., 2017) and limits myocyte damage during reoxygenation (Venkatesh et al., 2019). Failing mouse hearts display reduced proteolytic capacity due in large part to oxidative modifications of LonP1 which diminish its activity (Hoshino et al., 2014). The detrimental effects of prolonged UPR^mt^ induction (Gitschlag et al., 2016; Lin et al., 2016) and its paracrine effects on distal tissues (Berendzen et al., 2016; Shao et al., 2016) highlight the complexity of this mitochondrial QC mechanism and the need for additional studies.

## Role of Mitochondria in Cytosolic Protein Quality Control

Recent studies demonstrate that mitochondria can also directly contribute to cytosolic protein QC. For instance, induction of the UPR^mt^ is observed in various models of Alzheimer’s disease (AD) and is involved in reducing amyloid-β proteotoxicity (Sorrentino et al., 2017). Because the UPR^mt^ promotes the expression of mitochondrial chaperones and proteases, these observations indicate that this pathway might also support cytosolic proteostasis through import and degradation of pathogenic amyloid-β peptides. However, whether amyloid-β is imported into mitochondria is controversial (Petersen et al., 2008; Cenini et al., 2016). Petersen et al. (2008) reported that amyloid-β is transported into the mitochondrial matrix via the TOM complex, while Cenini et al. (2016) found that amyloid-β interferes with mitochondrial import of nuclear-encoded mitochondrial proteins. It is possible that amyloid-β can be imported into mitochondria before it forms aggregates; however, large proteotoxic aggregates are likely to interfere with various mitochondrial functions such as import. In addition, the UPR^mt^ has also been shown to promote a cytosolic chaperone response through the transcriptional regulator heat shock factor 1 that blunts the polyglutamine repeat protein (PolyQ) aggregation accompanying Huntington’s disease (Kim et al., 2016; Labbadia et al., 2017). Thus, the recent reports of UPR^mt^-mediated cardioprotection during pressure overload and ischemia may be applicable to protetotoxic cardiac diseases such as desmin-related cardiomyopathy and transthyretin amyloidosis.

**FIGURE 3:**
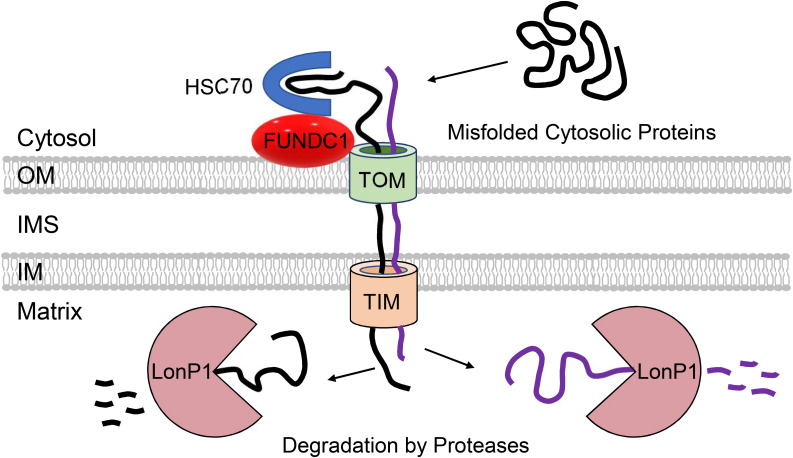
Participation of mitochondria in regulation of cytosolic proteostasis. Mitochondria contribute to cytosolic protein quality control through the uptake of aggregate-prone misfolded proteins via the TOM and TIM import machinery. FUNDC1 and HSC70 can facilitate the import of proteins into mitochondrial matrix where they are degraded by LonP1 protease.

A study by Ruan et al. (2017) demonstrated that in yeast, cytosolic proteins prone to aggregation are indeed imported into mitochondria via the TOM complex where they are subsequently degraded by the LonP1 protease. Although import of misfolded proteins at baseline is minimal, proteotoxic stress by heat-shock significantly increases mitochondrial import of cytosolic proteins. Importantly, loss of mitochondrial membrane potential abrogates this adaptive response, exacerbating cytosolic aggregation. This suggests that this potential novel QC pathway can compensate and reduce the load of aggregated proteins in the cytosol when cytosolic degradation is compromised (Figure 3). This study also noted that mitochondrial uptake of an aggregate-prone protein also occurs in human epithelial cells, but they did not examine effects on cell viability and proteostasis. The uptake of aggregate-prone proteins by mitochondria in mammalian cells was recently confirmed by another study. This group reported that proteosomal inhibition leads to the import of misfolded proteins where they are degraded in the matrix by LonP1 (Li et al., 2019). Interestingly, the OMM protein FUNDC1 was found to facilitate the mitochondrial translocation and import of UPS substrates through its interaction with the cytosolic chaperone HSC70 (Li et al., 2019) (Figure 3). As discussed below, FUNDC1 is also a known regulator of mitophagy. The authors also found that excessive uptake of misfolded proteins interferes with mitochondrial function and contributes to cellular senescence (Li et al., 2019). Excessive import of misfolded cytosolic proteins into the matrix is clearly toxic to mitochondria, but whether this led to activation of the UPR^mt^ still needs to be investigated. Mitochondrial import of cytosolic proteins might function as an important QC mechanism to ensure cellular proteostasis. However, whether this pathway is relevant in metabolically active tissues such as the heart remains to be investigated. It will be important to thoroughly evaluate the relevance of these QC mechanisms in cardiac myocytes considering the differences between their mitochondrial network, energetic demands, and regenerative potential relative to the various systems employed in the aforementioned investigations.

## Selective Elimination of Mitochondria

Mitochondrial autophagy (mitophagy) serves as a QC mechanism to remove entire damaged organelles that cannot be repaired by smaller scale stress responses such as the UPR^mt^ or UPS. Mitophagy targets dysfunctional mitochondria for autophagosomal engulfment and lysosomal degradation to ensure their elimination prior to cytotoxicity. The primary pathways involved in eliminating mitochondria are the PTEN-induced putative kinase 1 (PINK1)/Parkin pathway and mitophagy receptors in the OMM. The detailed molecular mechanisms of PINK1/Parkin- and mitophagy receptor-mediated mitochondrial elimination have previously been reviewed in detail (Gustafsson and Dorn, 2018; Pickles et al., 2018). The conditions that activate these different pathways are under intense investigation and the two pathways appear to function under both distinct and overlapping conditions, making it a challenging area of research.

**FIGURE 4:**
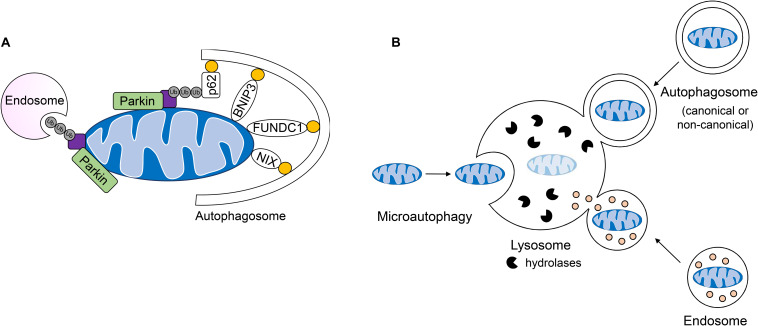
Lysosomal-mediated degradation of mitochondria. **(A)** Mitochondria are labeled by the E3 ubiquitin ligase Parkin for mitochondrial autophagy (mitophagy) or removal by endosomes. Engulfment of ubiquinated cargo involves autophagy adaptors such as p62/SQSTM1 which link ubiquinated cargo to the autophagosome through LC3/GABARAP interaction. Outer mitochondrial membrane mitophagy receptors BNIP3, BNIPL3L/NIX, and FUNDC1 facilitate the degradation of whole organelles through direct interaction with LC3/GABARAP. **(B)** Mitochondria can be directly taken up by the lysosome, engulfed by single-membraned endosomes or double-membraned autophagosomes which fuse with the lysosome for the degradation of cargo.

Most studies to date in the heart have focused on the PINK1/Parkin pathway, which is involved in the selective removal of damaged mitochondria during stress. In this pathway, the serine/threonine kinase PINK1 is constitutively imported into healthy mitochondria, where it is cleaved by proteases in the intermembrane space and then returned to the cytosol for proteasomal degradation (Jin et al., 2010; Greene et al., 2012; Yamano and Youle, 2013). When damaged or dysfunctional mitochondria lose their membrane potential, import and degradation of PINK1 are abrogated, resulting in its accumulation on the OMM (Matsuda et al., 2010; Narendra et al., 2010; Vives-Bauza et al., 2010). Interestingly, although loss of mitochondrial membrane potential has been reported to be the primary mechanism for activation of this pathway, one study reported that excessive protein misfolding in the matrix of respiring mitochondria with intact membrane potential also leads to inhibition of PINK import and activation of mitophagy (Jin and Youle, 2013). At the OMM, PINK1 recruits the E3 ubiquitin ligase Parkin (Narendra et al., 2010), which in turn, proceeds to ubiquitinate numerous proteins in the OMM (Sarraf et al., 2013). This leads to recruitment of the autophagosome machinery and sequestration of the mitochondria (Figure 4A). The PINK1/Parkin pathway plays an important role in the heart and many studies have reported on its importance in repair and adaptation to stress. For instance, PINK1/Parkin-mediated mitophagy is activated during pressure-overload (Billia et al., 2011; Shirakabe et al., 2016), following myocardial infarction (MI) (Kubli et al., 2013), and in I/R (Siddall et al., 2013). A recent study also discovered that mitophagy is increased in hearts of mice fed a high-fat diet and that Parkin deficiency results in increased cardiac dysfunction (Tong et al., 2019). Overall, these studies have confirmed a key role of PINK1/Parkin-mediated mitophagy in the adult heart where dysfunctional mitochondrial must be eliminated to prevent loss of myocytes and cardiac dysfunction.

Voltage-dependent anion channels (VDACs), which regulate metabolite and ion transport across the OMM, are also involved in regulating PINK1/Parkin-dependent mitophagy. It has been reported that the VDACs are responsible for recruiting Parkin to depolarized mitochondria (Sun et al., 2012). There are three VDAC genes (VDAC1, VADC2, and VDAC3) and only simultaneous knockdown or deletion of all three VDACs abrogates mitochondrial Parkin translocation (Sun et al., 2012). In addition, the 18 kDa outer mitochondrial translocator protein TSPO disrupts mitophagy through its interaction with VDAC1 (Gatliff et al., 2014). This study found that TSPO does not affect Parkin translocation to mitochondria, but prevents Parkin from ubiquitinating its substrates. How TSPO inhibits Parkin activity is currently unknown. Interestingly, heart failure is associated with increased TSPO expression and cardiac-specific TSPO KO mice are protected from pressure-overload-induced cardiac remodeling and dysfunction (Thai et al., 2018). This study also found that while mitophagy is impaired in wild-type myocytes after 8 weeks of pressure overload, mitophagy is preserved in TSPO-deficient myocytes (Thai et al., 2018). Collectively, these findings point to important functions for TSPO and VDAC in regulating Parkin-dependent mitophagy in cardiac myocytes.

Interestingly, PINK1/Parkin-mediated clearance of damaged mitochondria in tissues also limits inflammation by preventing overactivation of the inflammasome. These cytosolic multimeric protein complexes function as sensors of infectious microbes and molecules derived from pathogens. Once activated, the inflammasome activates caspase-1 and induces inflammation in tissues (Malik and Kanneganti, 2017). Unfortunately, overactivation of the inflammasome can lead to excessive inflammation and tissue damage. Because mitochondria are descendants of bacteria, release of mtDNA into cytosol from damaged mitochondria will also activate the inflammasome. Induction of mitophagy to eliminate these mitochondria prior to release of mtDNA prevents inflammasome activation (Zhong et al., 2016). Early studies on Parkin in the heart led to the observation that Parkin-deficient mice are more sensitive to endotoxins and develop increased mitochondrial damage and contractile dysfunction after endotoxin exposure compared with wild-type mice (Piquereau et al., 2013). Subsequent studies have reported that mice with enhanced PINK1/Parkin-mediated mitophagy in hearts render these mice more resistant to cardiac dysfunction and alleviate inflammation and fibrosis after endotoxin exposure (Sun et al., 2018; Essandoh et al., 2019). Interestingly, another study focused on investigating why heart failure is accelerated after an MI in patients with Type 2 diabetes mellitus discovered a defect in myocardial mitophagy in these patients that correlates with increased release of mitochondrial DNA and hyperactivation of the NLRC4 inflammasome (Devi et al., 2017). Overall, these studies demonstrate an important link between mitophagy and inflammation in the heart.

Receptor-mediated mitophagy is also important in the heart and differs from the PINK1/Parkin pathway in that ubiquitination and adaptor proteins are not needed for recognition of cargo by the autophagosome (Gustafsson and Dorn, 2018). Rather, OMM-anchored mitophagy receptors such as BNIP3, BNIP3L/NIX, and FUNDC1 directly tether the mitochondrion to the autophagosome membrane via their interaction with LC3 and GABARAP to facilitate its clearance (Gustafsson and Dorn, 2018) (Figure 4A). The conditions that induce activation of receptor-mediated mitophagy are less clear. However, there is strong evidence that while PINK1/Parkin-dependent mitophagy requires mitochondrial membrane depolarization, receptor-mediated mitophagy is often induced by hypoxia. Many of the mitophagy receptors are induced during hypoxic conditions (Chen et al., 2017; Esteban-Martínez and Boya, 2018). For instance, BNIP3 and NIX are hypoxia-inducible pro-apoptotic members of the BH3-only BCL-2 family with 56% sequence homology (Zhang and Ney, 2009). Both BNIP3 and NIX have dual roles in inducing apoptosis and mitophagy, which has made it challenging to dissect their functions in cells. Overexpression of BNIP3 and NIX in cells, including myocytes, leads to activation of both mitophagy and cell death (Regula et al., 2002; Chen et al., 2010). However, studies indicate that their primary function is to induce mitophagy but that during overwhelming stress and mitochondrial damage, they turn on their pro-death function. This is supported by the fact that BNIP3-deficiency in mice leads to attenuated myocyte apoptosis and preserves contractile performance in models of I/R injury (Diwan et al., 2007) and doxorubicin cardiotoxicty (Dhingra et al., 2014). This suggests that BNIP3 contributes to cell death and cardiac injury under these conditions. However, simultaneous deletion of NIX and BNIP3 in hearts leads to accelerated accumulation of dysfunctional mitochondria in myocytes with age (Dorn, 2010), implicating these proteins as key regulators of normal mitochondrial turnover in hearts in the absence of stress.

FUNDC1 is another mitophagy receptor that facilitates hypoxia-induced mitochondrial clearance (Chen et al., 2017). FUNDC1 activity is regulated by phosphorylation through several different kinases. Dephosphorylation by the mitochondrial phosphatase PGAM5 is required for its activation and interaction with LC3 to induce mitophagy (Chen et al., 2014). Inhibition of FUNDC1 seems to be a major factor underlying myocardial I/R injury. Several kinases, including MST1 (Yu et al., 2019), CK2α (Zhou et al., 2018), and RIPK3 (Zhou et al., 2017), have been reported to inhibit FUNDC1-mediated mitophagy during I/R. For instance, Zhou et al. (2018) reported that upregulation of CK2α during myocardial I/R inhibits FUNDC1-mediated mitophagy and results in accumulation of dysfunctional mitochondria, opening of the mitochondrial permeability transition pore, and activation of cell death. What happens to other mitophagy receptors under these conditions and their relationship with FUNDC1 activity remain to be investigated.

Recent observations of mitochondrial clearance in cells lacking key canonical autophagy regulators *Atg5* and *Atg7* have led to the identification of alternative mechanisms of mitochondrial degradation in cells. For instance, a non-canonical autophagy pathway have been identified where Rab9-positive double-membrane vesicles derived from the trans-Golgi can engulf mitochondria which are then delivered to lysosomes for degradation (Nishida et al., 2009). This alternative autophagy pathway plays a role in clearing mitochondria during starvation and in myocardial ischemia (Hirota et al., 2015; Saito et al., 2019). It has also been reported that dysfunctional mitochondria can be sequestered in Rab5-positive early endosomes for subsequent delivery to the lysosome for degradation (Hammerling et al., 2017a, b). The uptake of depolarized mitochondria into early endosomes is dependent on Parkin-mediated ubiquitination of mitochondrial proteins which are then recognized by the ESCRT machinery (Figure 4A). These ESCRT complexes facilitate capture and delivery of mitochondria into the endosomal lumen. Interestingly, the mitophagy receptor BNIP3 can also utilize the endosomal pathway for mitochondrial elimination (Hammerling et al., 2017b), but whether this requires ubiquitination and the ESCRT machinery is currently unclear. Although BNIP3-mediated sequestration of mitochondria into Rab5-positive endosomes is independent of Parkin, it is possible that BNIP3 still utilizes a resident E3 ubiquitin ligase to facilitate uptake into endosomes. In addition to the endosome pathway, microautophagy represents another form of non-canonical autophagy that involves the direct uptake of cargo into lysosomes; however, this process is not well characterized (Gustafsson and Dorn, 2018). It has been reported that mitochondria can be directly delivered to lysosomes in mammalian cells and that GAPDH regulates their engulfment (Hwang et al., 2015). This mode of mitochondrial degradation has been reported to be inhibited in myocardial I/R injury (Yogalingam et al., 2013) and in Huntington’s disease (Hwang et al., 2015). Thus, it is likely that GAPDH-mediated mitochondrial engulfment by the lysosome represents another major mechanism of mitochondrial QC, in particular when formation of autophagosomes is reduced or compromised. In sum, whole mitochondria are degraded by the lysosome through direct fusion, or engulfment in double and single-membraned autophagosomes and endosomes, respectively (Figure 4B).

## Coordination Between Mitochondrial Elimination and Biogenesis

Mitophagy is tightly coordinated with mitochondrial biogenesis to appropriately balance the degradation and formation of new organelles. Baseline mitophagy involving removal of dysfunctional and aged organelles is always accompanied by biogenesis for replacement of mitochondria that were degraded. This continuous balance between degradation and synthesis is particularly important in highly energetic cells such as cardiac myocytes and neurons that rely on mitochondria for function. Similarly, during stress, when a larger portion of mitochondria are eliminated, there is a coordinated activation of mitochondrial biogenesis. In addition to inducing mitophagy, Parkin simultaneously induces mitochondrial biogenesis by indirectly activating the PPARG coactivator 1 alpha (PGC-1α), a master regulator of mitochondrial biogenesis in the heart (Lehman et al., 2000; Arany et al., 2005). Parkin promotes the ubiquitination and proteasomal degradation of PARIS, a transcriptional repressor of PGC-1α (Shin et al., 2011). Parkin deficiency leads to increased levels of PARIS and reduced mitochondrial mass in dopaminergic neurons (Stevens et al., 2015). In skeletal muscle injury (Vainshtein et al., 2015) and exercise (Erlich et al., 2018) models, PGC-1α exerts a positive feedback loop to enhance mitophagy through the induction of TFEB, a transcriptional regulator of the lysosomal machinery. Similarly, the metabolic sensor and autophagy-promoting kinase AMPK has been shown to phosphorylate PGC-1α (Jäger et al., 2007) and induce downstream PGC-1α expression (Cantó et al., 2009). Apart from PGC-1α and AMPK, emerging evidence suggests that the transcriptional regulator of antioxidant gene expression nuclear factor, erythroid 2 like 2 (Nfe2l2/Nrf2) coordinates both mitophagy and mitochondrial biogenesis. Nrf2 promotes the expression of biogenesis genes Nrf-1 (Merry and Ristow, 2016) and Tfam (Wu et al., 2016) as well as mitophagy factors p62/SQSTM1 (Jain et al., 2010) and DCT-1 (BNIP3 ortholog) (Palikaras et al., 2015). However, while Nrf2 protects the heart from ischemic injury (Zhang et al., 2010) and pressure overload (Li et al., 2009), constitutive myocardial Nrf2 activation paradoxically promotes pathophysiology through proteotoxicity (Rajasekaran et al., 2011; Shanmugam et al., 2020). Thus, a closer examination into Nrf2-mediated mitochondrial QC in cardiac myocytes is warranted. Mitochondrial uptake of misfolded proteins was recently linked to increased mitophagy and biogenesis in a human cell model of ribosomal mistranslation (Shcherbakov et al., 2019). Therefore, mitochondrial import, clearance, and biogenesis may integrate as a coordinated stress response against proteotoxicity; however, more studies are needed to examine the potential for this cross-talk in cardiac myocytes.

In contrast, proper erythrocyte maturation necessitates bulk degradation of mitochondria in the absence of compensatory biogenesis, and this is achieved through the mitophagy receptor NIX (Sandoval et al., 2008). However, how mitochondrial biogenesis is suppressed in these cells during mitophagy is unclear, but could be due to the mechanism by which mitochondria are cleared (Parkin vs. NIX). Although a clear link exists between Parkin and activation of mitochondrial biogenesis, a similar link between NIX and mitochondrial biogenesis has not been reported. Clearly, coordination between mitochondrial degradation and biogenesis varies according to cell-type and physiological context.

## Mitochondrial-Derived Vesicles

**FIGURE 5:**
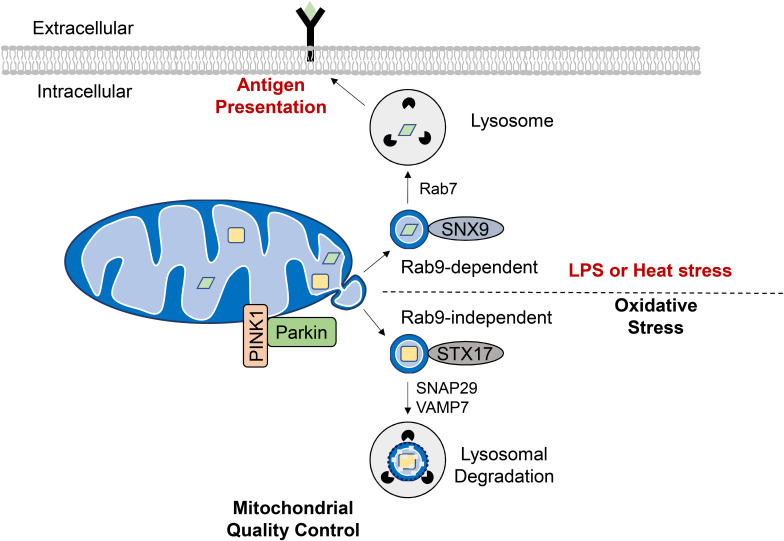
Formation of mitochondrial derived vesicles (MDVs) as a quality control mechanism or antigen presentation. Generation of MDVs in response to oxidative stress requires PINK1 and Parkin, while STX17, SNAP29, and VAMP7 are required for their fusion with the lysosome. These vesicles contain oxidized cargo that are degraded in the lysosomes. MDVs are also formed in response to LPS exposure or heat stress and require the Rab7, Rab9, and SNX9. The mitochondrial antigens are processed in the lysosome for subsequent presentation on MHC class I molecules at the cell surface.

To avoid eliminating the entire mitochondrion, damaged proteins and lipids can be selectively incorporated into vesicles that bud off from the OMM. These mitochondrial-derived vesicles (MDVs) have been found to be enriched with oxidized protein cargo suggesting that their formation is an additional mitochondrial QC mechanism (Soubannier et al., 2012b). Interestingly, ROS-induced MDV formation precedes mitophagy, with vesicles forming as early as 2 h following antimycin A treatment to inhibit mitochondrial respiration (McLelland et al., 2014). The MDVs are 70–150 nM in diameter and ultrastructural analysis has confirmed that they can be either double-membrane vesicles containing matrix cargo or single-membraned containing outer membrane proteins (Soubannier et al., 2012b). Incorporation of MDV cargo appears to be highly specific as the origin of ROS (e.g., intra vs. extraorganellar) dictates whether MDVs contain oxidized cargo from the inner compartments or OMM (Soubannier et al., 2012b). A subset of MDVs is targeted for lysosomal degradation through the endosomal system (Soubannier et al., 2012a) (Figure 5). The formation of these MDVs during mitochondrial stress requires PINK1 and Parkin, and Parkinson’s disease-associated Parkin mutants are defective in MDV formation (McLelland et al., 2014, 2016). Their formation is independent of mitochondrial fission (Soubannier et al., 2012a) and does not require Atg5 and Beclin1 (McLelland et al., 2014) suggesting an upstream mechanism that is distinct from general autophagy. Also, the SNARE protein syntaxin-17 (STX17) is recruited to the MDVs where it forms a complex with SNAP29 and VAMP7 to facilitate their trafficking and endolysosomal fusion in a homotypic fusion and vacuole protein sorting (HOPS) tethering complex-dependent manner (McLelland et al., 2016).

Interestingly, Matheoud et al. identified a distinct set of MDVs that function in mitochondrial antigen presentation (Figure 5). Antigen presentation plays a role in establishing immune tolerance and is important in limiting autoimmunity (Matheoud et al., 2016). This study found that exposure to heat stress or LPS leads to transportation of mitochondrial proteins in MDVs to lysosomes for subsequent presentation on MHC class I molecules at the cell surface. The formation of these MDVs requires Rab7, Rab9, and sorting nexin 9 (SNX9) and is negatively regulated by PINK1/Parkin (Matheoud et al., 2016). Moreover, the fact that MDV-mediated mitochondrial antigen presentation requires Rab9 (Matheoud et al., 2016) while oxidative stress-induced lysosomal degradation of MDVs is Rab-9-independent (McLelland et al., 2014) indicates that distinct MDV transport mechanisms exist for diverse cellular stimuli. Indeed, peroxisomal delivery represents an alternative fate for MDV cargo and this process appears to uniquely require the vacuolar sorting protein VPS35 (Braschi et al., 2010). Continued investigation is needed to elucidate the key regulators of MDV formation, trafficking and degradation as a QC mechanism, and to differentiate this process from other homeostatic routes of MDV transport.

Little is still known about the tissue-specific role for MDVs and their relevance in human disease; however, this level of mitochondrial QC is likely to be critical for cell types with a high energetic demand that cannot afford to clear large portions of the mitochondrial network via mitophagy. Indeed, MDV formation appears to be an active process in the heart. Cadete et al. demonstrated that MDVs are formed in the H9c2 myoblast cell-line and murine hearts at baseline. MDVs carrying OMM and matrix proteins are observed in H9c2 cells under baseline conditions and vesicle biogenesis is significantly increased by ROS-inducing stimuli. Transmission electron microscopy of mouse hearts after exposure to antimycin A show formation of both single and double-membraned MDVs. Importantly, while hearts from control mice show MDV budding in the basal state, doxorubicin-treated mice depict a significant increase in myocardial MDV accumulation indicating that MDV formation contributes to mitochondrial quality during physiologic and pathological conditions *in vivo* (Cadete et al., 2016). The identification of MDVs adds yet another layer of mitochondrial QC in cardiac myocytes. It is likely that this process represents the first level of defense against mitochondrial damage; however, myocyte-specific effectors remain elusive as these studies employed wild-type mice without individual silencing of previously identified MDV regulators. As Cadete and colleagues utilized a doxorubicin model, future studies should aim to assess the temporal contribution of lysosomal MDV degradation in myocardial ischemia and reperfusion injury, and elucidate cross-talk with other QC pathways discussed above.

## Therapeutic Potential of Enhancing Proteostasis and Mitochondrial Quality Control

The fact that damaged mitochondria are consistently reported to accumulate in the diseased myocardium suggests that strategies designed to promote mitochondrial QC may hold therapeutic potential. Both general and mitochondrial autophagy transiently increase during pathological cardiac stress, but these compensatory mechanisms are ultimately exhausted at the point of overt heart failure (Shirakabe et al., 2016). Moreover, cardiac aging has been linked to diminished autophagy and mitophagy. The aged myocardium is often burdened with excessive reactive oxygen and nitrogen species which exert an inhibitory effect on the function of autophagy related proteins (Frudd et al., 2018) and Parkin (Meng et al., 2011). Proteotoxic aggregates also promote oxidative stress (Tanase et al., 2016) and can overwhelm UPS and lysosomal machinery in cardiac myocytes (Pan et al., 2017; Xu et al., 2020). Thus, enhancing lysosomal degradation of proteotoxic species and dysfunctional mitochondria promotes cardiac longevity.

Spermidine and urolithin A are two naturally occurring compounds recently reported to induce autophagy and mitophagy in pre-clinical models. Interestingly, “late-in-life” spermidine supplementation to 18-month old mice attenuates the pathological cardiac remodeling and dysfunction normally seen at 23 months in rodents (Eisenberg et al., 2016). Spermidine is also cardioprotective in high salt diets suggesting that this bioavailable compound may translate to human aging and congestive heart failure (Eisenberg et al., 2016). While spermidine-mediated protection is Atg5-dependent (Eisenberg et al., 2016), urolithin A appears to be a more selective activator of mitophagy as urolithin A-mediated increases in aged nematode mobility and lifespan require PINK1 (Ryu et al., 2016). Urolithin A administration to 24-month old mice increases skeletal muscle strength and exercise performance (Ryu et al., 2016). While this study did not examine the cardiac-specific effects, a different group recently reported diminished myocardial I/R injury in adult mice pre-treated with urolithin A (Tang et al., 2017). In addition to spermidine and urolithin A, the mTOR inhibitor rapamycin has been tested as a cardioprotective agent for the age-associated decline in myocardial autophagy. A 3-month rapamycin regimen in 24-month old mice reverses age-induced cardiac hypertrophy and dysfunction (Flynn et al., 2013). At the molecular level, the cardioprotective effects of rapamycin have been attributed to decreased protein oxidation and ubiquitination (Dai et al., 2014) as well as enhanced mitochondrial biogenesis (Chiao et al., 2016). Taken together, these observations highlight the importance of targeting proteostasis and mitochondrial QC in therapeutic interventions for cardiac aging.

Caloric restriction is also a well-documented inducer of autophagy in mammalian cells (Escobar et al., 2019), and has been reported to reduce age-associated cardiovascular disease (Colman et al., 2009) and increase the lifespan (Pifferi et al., 2018) of primates. The heart is sensitive to nutrient availability as a single overnight fast is sufficient to stimulate autophagy in adult mouse hearts (Andres et al., 2016). Moreover, long-term gradual caloric reductions (e.g., 20–60% deficit for 4 weeks) also promote myocardial autophagic flux (Finckenberg et al., 2012; Chen et al., 2013). Considering the cost, availability, and multifaceted benefits of dietary intervention on human health, caloric restriction may provide the greatest translational value of the aforementioned therapies. Indeed, a recent randomized control trial of alternate day fasting in humans reports improved heart rate, blood pressure, and Framingham Risk Score, suggesting enhanced cardiovascular health (Stekovic et al., 2019). Although caloric intake was reduced in this study (Stekovic et al., 2019), time-restricted feeding, often referred to as intermittent fasting, elicits cardiometabolic benefits even when total caloric intake is maintained (Hatori et al., 2012). The fact that intermittent fasting modulates the expression of electron transport chain and protein folding machinery in a manner that reduces cardiac aging suggests that feeding rhythms are tightly linked to mitochondrial QC and proteostasis (Gill et al., 2015). This is further supported by recent data demonstrating that an intermittent fasting regimen during advanced protein aggregation cardiomyopathy restores autophagic flux and cardiac function in mice (Ma et al., 2019). In summary, caloric restriction or intermittent fasting holds therapeutic promise in part through protein and mitochondrial QC downstream of enhanced autophagy.

Intermittent fasting increases circulating ketone levels in humans (Jamshed et al., 2019). As an alternative fuel substrate, ketone bodies have recently received significant attention for their protective affects in a variety of neurological disorders such as AD (Kashiwaya et al., 2013), epilepsy (Kovács et al., 2019), and anxiety (Ari et al., 2017). During heart failure, diminished fatty acid oxidation results in a compensatory increase in myocardial ketone oxidation (Aubert et al., 2016). This response appears to be adaptive as genetically modified mice incapable of catabolizing 3-hydroxybutyrate (3OHB) display worsened contractile dysfunction and cardiac remodeling following transverse aortic constriction combined with a small apical MI (Horton et al., 2019). Importantly, exogenous 3OHB infusion in an established canine model of congestive heart failure preserves cardiac output and ejection fraction while attenuating left ventricular hypertrophy and chamber dilation (Horton et al., 2019). While the effects of ketone metabolism on mitochondrial QC are limited, Thai et al. (2019) recently demonstrated that 24 h of ketone treatment *in vitro* promotes Parkin-dependent mitophagy in young (2.5-month) and aged (2.5-year) rabbit ventricular myocytes. However, aged cardiac myocytes subjected to heart failure are not protected by β-hydroxybutyrate. Rather, ketone administration in these cells aggravates the incidence of Parkin aggregation at depolarized mitochondria (Thai et al., 2019). Interestingly, combining β-hydroxybutyrate with the mitochondrial fusion promoting peptide TAT-MP1^Gly^ (Franco et al., 2016) promotes mitochondrial QC in aged and failing myocytes, suggesting that a sufficient level of mitochondrial dynamics is required for the protective effects of ketone supplementation in heart failure (Thai et al., 2019). Combined activation of fusion and mitophagy is also cardioprotective in a model of angiotensin-II-induced cardiomyocyte injury (Xiong et al., 2019). These recent observations indicate that ketones exert a beneficial effect on cardiac myocytes through bioenergetic efficiency (Horton et al., 2019) and mitochondrial QC (Thai et al., 2019). However, more studies are needed to differentiate the effects from fasting-induced ketosis and exogenous ketone supplementation. Because a long-term ketogenic diet increases mitochondrial ROS and reduces respiratory control ratio in the skeletal muscle (Kephart et al., 2017), it will be important to optimize dose and duration before translating to the clinic.

While it is clear that promoting autophagy through caloric restriction or intermittent fasting is beneficial for cardiac aging, more studies are needed to address mitochondria-specific effects of these therapeutic strategies. The removal of mitochondria represents only one aspect of the autophagy pathway. Furthermore, little is known about the relative contributions of various mitophagy receptors and the endosomal-mediated pathway across various cardiac disease settings. As lysosomal degradation likely represents the last stage of QC, a deeper understanding of lower scale responses such as the mitochondrial UPR and MDV’s will be critical for more personalized approaches. Although much of the mechanistic underpinnings remain elusive, the pharmacological UPR^mt^ activator nicotinamide riboside (NR) has been shown to protect against pressure overload (Smyrnias et al., 2019) and dilated cardiomyopathy (Diguet et al., 2018) in mice, thereby justifying an early phase clinical trial in human heart failure patients (NCT03727646). However, reports leading up to this trial have focused on the reversal of mitochondrial protein hyperacteylation (Lee et al., 2016; Airhart et al., 2017; Walker and Tian, 2018), rather than UPR^mt^ induction. Moving forward, it will be important to elucidate the relationship between these processes as it relates to the efficacy of NR. There is also evidence that excessive autophagy induction (Gao et al., 2018) and Parkin expression (Woodall et al., 2019) are maladaptive in the heart. As such, fine-tuning safe thresholds for bulk degradation, and continued investigation into novel regulators of alternative pathways is warranted.

## Conclusion

Pathophysiological stress often damages mitochondria in myocytes which are vital for the heart’s contractile activity. Therefore, continuous monitoring and repair of mitochondria are needed to maintain a healthy mitochondrial population in cells. Multiple levels of mitochondrial QC exist both at the protein and organelle level. Here, we have reviewed the intricate pathways that coordinate mitochondrial quality in cells and how they are altered in the diseased heart. First, because the majority of mitochondrial proteins are encoded in the nucleus, significant monitoring of mitochondrial precursor proteins is needed during their cytosolic translation and import. The UPS shapes the mitochondrial proteome through steady-state turnover of mitochondrial precursors to ensure an appropriate stoichiometry between nuclear and mitochondrially encoded proteins and their proper localization (Margineantu et al., 2007; Radke et al., 2008; Azzu and Brand, 2010; Bragoszewski et al., 2013, 2015). Second, mitochondria contain resident chaperones and proteases to ensure QC within the mitochondria (Lau et al., 2012; Bulteau et al., 2017). Third, excessive levels of misfolded proteins in the mitochondrial matrix or a mito-nuclear protein imbalance activates a conserved UPR^mt^ which functions to selectively induce a transcriptional response aimed at restoring mitochondrial proteostasis (Zhao et al., 2002; Shpilka and Haynes, 2018). A closer examination into these processes reveals an inextricable link between mitochondrial QC and cytosolic proteostasis. More recently, mitochondria themselves have been found to participate in general protein QC through the import and degradation of misfolded cytosolic proteins (Ruan et al., 2017; Li et al., 2019). In the event that the mitochondria cannot be repaired, myocytes have the option of either eliminating damaged mitochondrial components via MDVs (McLelland et al., 2014, 2016), or by removing the entire organelle through mitophagy (Gustafsson and Dorn, 2018). Elimination of the entire mitochondria is likely a last resort response because it requires the cell to replace the mitochondrion. Continued investigations into the molecular drivers of mitochondrial quality have the potential to elucidate novel interventions for general the proteostatic stress seen during myocardial ischemia, pressure overload, and protein aggregation cardiomyopathies (Hofmann et al., 2019). Collectively, these mitochondrial QC pathways represent essential adaptive responses in cardiac myocytes, and fruitful avenues for the development of novel therapies against cardiovascular diseases. Once a better understanding of the regulators and relationships between the various QC pathways is gained, we will hopefully be able to translate this knowledge into improved treatments for disease.

## Author Contributions

Both authors contributed to the content of this article and approved of its submission.

## Conflict of Interest

The authors declare that the research was conducted in the absence of any commercial or financial relationships that could be construed as a potential conflict of interest.
